# Combining genomic sequencing methods to explore viral diversity and reveal potential virus-host interactions

**DOI:** 10.3389/fmicb.2015.00265

**Published:** 2015-04-10

**Authors:** Cheryl-Emiliane T. Chow, Danielle M. Winget, Richard A. White, Steven J. Hallam, Curtis A. Suttle

**Affiliations:** ^1^Department of Earth, Ocean, and Atmospheric Sciences, University of British ColumbiaVancouver, BC, Canada; ^2^Department of Microbiology and Immunology, University of British ColumbiaVancouver, BC, Canada; ^3^Integrated Microbial Biodiversity Program, Canadian Institute for Advanced ResearchToronto, ON, Canada; ^4^Graduate Program in Bioinformatics, University of British ColumbiaVancouver, BC, Canada; ^5^Department of Botany, University of British ColumbiaVancouver, BC, Canada

**Keywords:** microbial ecology, marine virus, metagenomics, fosmids, virome, reference genome, single cell genomics

## Abstract

Viral diversity and virus-host interactions in oxygen-starved regions of the ocean, also known as oxygen minimum zones (OMZs), remain relatively unexplored. Microbial community metabolism in OMZs alters nutrient and energy flow through marine food webs, resulting in biological nitrogen loss and greenhouse gas production. Thus, viruses infecting OMZ microbes have the potential to modulate community metabolism with resulting feedback on ecosystem function. Here, we describe viral communities inhabiting oxic surface (10 m) and oxygen-starved basin (200 m) waters of Saanich Inlet, a seasonally anoxic fjord on the coast of Vancouver Island, British Columbia using viral metagenomics and complete viral fosmid sequencing on samples collected between April 2007 and April 2010. Of 6459 open reading frames (ORFs) predicted across all 34 viral fosmids, 77.6% (*n* = 5010) had no homology to reference viral genomes. These fosmids recruited a higher proportion of viral metagenomic sequences from Saanich Inlet than from nearby northeastern subarctic Pacific Ocean (Line P) waters, indicating differences in the viral communities between coastal and open ocean locations. While functional annotations of fosmid ORFs were limited, recruitment to NCBI's non-redundant “nr” database and publicly available single-cell genomes identified putative viruses infecting marine thaumarchaeal and SUP05 proteobacteria to provide potential host linkages with relevance to coupled biogeochemical cycling processes in OMZ waters. Taken together, these results highlight the power of coupled analyses of multiple sequence data types, such as viral metagenomic and fosmid sequence data with prokaryotic single cell genomes, to chart viral diversity, elucidate genomic and ecological contexts for previously unclassifiable viral sequences, and identify novel host interactions in natural and engineered ecosystems.

## Introduction

The long evolutionary history of viruses with cellular life is evident from the diseases they cause, such as influenza and AIDS, and also from the viral genes found in the genomes of cells. These relationships have their origins in viruses that infect bacteria, archaea and protists, all of which play a critical role in global nutrient and energy cycling and in maintaining functional ecosystems. Viruses affect the abundance and diversity of phytoplankton (e.g., Larsen et al., 2004), bacteria (e.g., Winter et al., 2004), and archaea (e.g., Andersson and Banfield, 2008), and consequently influence global biogeochemical cycles (Fuhrman, 1999; Wilhelm and Suttle, 1999; Suttle, 2007; Winget et al., 2011), and genome evolution (Shackelton and Holmes, 2004; Sharon et al., 2007). Despite our emerging understanding that viruses play important roles in the earth system, our knowledge of the distribution of viral genotypes, their dispersal among environments, their ecological niches, and the functions of most viral genes remain largely unknown (Brussaard et al., 2008).

Although advances in nucleic-acid technologies have greatly increased the rate and depth to which the genetic diversity and ecology of viral communities can be interrogated, the inferences drawn from sequence-based investigations are hampered by methodological biases and non-representative databases of viral sequences. These limitations are exacerbated by the ultra-low quantity of nucleic acids in viral particles, the enormous genetic diversity of viruses in nature, and the lack of relevant model systems across a breadth of viral taxonomic groups. For example, most of the sequenced and available dsDNA viral genomes are from tailed phages in the order *Caudovirales*, although these and related genotypes are not dominant in marine systems (e.g., Breitbart et al., 2002; Angly et al., 2006). Consequently, only a small proportion of viral metagenomic reads can be aligned with sequenced viral genomes and placed in a genomic context. Moreover, the majority of predicted viral open reading frames (ORFs) have no functional annotation (Angly et al., 2006; Williamson et al., 2012; Hurwitz and Sullivan, 2013; Hurwitz et al., 2015), leaving viral ecologists to wonder what most of this genetic material represents. In the absence of an abundance of and wider diversity of viral isolates and host systems, viral genomic information must be gleaned using alternative methods [e.g., large insert fosmid libraries (Garcia-Heredia et al., 2012; Mizuno et al., 2013b), sequencing of viral DNA extracted from pulsed-field gel electrophoresis bands (Ray et al., 2012), or single-virus genomics (Allen et al., 2011)]. Additionally, by targeting and sorting viral or host populations with flow cytometry, genomic data can be obtained for specific virus-host interactions (Deng et al., 2014; Martinez-Martinez et al., 2014). Mining cellular metagenomic and single-cell genome datasets has also unearthed new virus genomes and identified potential virus-host relationships (Anantharaman et al., 2014; Roux et al., 2014b) from previously uncultured hosts. These inferred virus-host interactions not only reveal a past virus encounter and subsequent infection of a host organism but also indicate the potential for genetic exchange during the infection cycle that can drive consequent effects on the metabolic status and rates of the infected host. When viral genomic data can be linked to a specific host organism, it becomes possible to study virus-host interactions within natural or engineered ecosystems and place “viral dark matter” into an ecological context.

In this study, viral fosmid and metagenomic sequences combined with bacterial single-cell genomes (SAGs) were interrogated with the goals of placing viral metagenomic sequence data into a genomic context and revealing host-virus interactions. Large-insert fosmid sequences (~35 kb) served as proxies for partial or nearly-complete dsDNA viral genomes; for the most abundant viruses, typical genome sizes range from about 29 to 69 kb in seawater (Steward et al., 2000). Saanich Inlet, British Columbia was used as a model site as it is a fjord that undergoes seasonal cycles of stratification and renewal that dynamically alter the oxygenation status of the water column (Anderson and Devol, 1973). During peak stratification, a redoxcline develops with anoxic and sulfidic conditions prevailing in the deep basin waters (200 m). Expansion of areas of low oxygen concentration are becoming of increasing concern worldwide (Wright et al., 2012), yet viruses and their roles in these low oxygen marine environments remain poorly studied. Comparison of metagenomic and fosmid sequences show that viruses in Saanich Inlet were distinct from those in other environments and identified putative viruses infecting marine thaumarchaea and members of the bacterial SUP05 clade.

## Materials and methods

### Sample collection

Sample collection was carried out on board the *MSV John Strickland* in Saanich Inlet, British Columbia at station S3 (48° 35′ 30.0012″N, 123° 30′ 21.9996″W). A sill at the fjord mouth prevents mixing and oxygenation except for deep water renewal events in early September and after unusually strong storms (Anderson and Devol, 1973), which leads to hypoxic condition below the mixed layer for most of the year. Approximately 20 L of seawater was collected monthly by wire-mounted Niskin bottles at 10 m and 200 m depth intervals from April 2007 to April 2010. Seawater was filtered through a 0.22−μm pore-size Sterivex filter (Millipore) to remove the cellular fraction. Viruses were concentrated by tangential flow ultrafiltration through a 30-kDa molecular-weight cutoff cartridge (Prep-Scale 2.5, Millipore) to a final volume between 250 and 500 mL (Suttle et al., 1991), and stored at 4°C until further processing.

Ten-mL subsamples of viral concentrates (VCs) from either 10 m or 200 m were combined into composite mixes to create fosmid libraries for each of summer, fall, and winter and a mix of 32 VCs spanning 3 years was used for metagenomic sequencing (Table S1). VC mixes were filtered again through 47 mm diameter, 0.22−μm pore-size filters after mixing (Type PVDF: polyvinylidene fluoride, Millipore) and then further concentrated to between 1 and 2 mL using a 30 kDa molecular-weight cutoff Centricon filter by spinning at 3000 rpm (~825 × g) for 8–10 min at 10°C in a benchtop centrifuge.

### DNA extraction and sequencing library preparation

#### Viral fosmid libraries

Within 48 h of Centricon concentration, viral DNA was extracted from each VC mix as follows. First, free DNA and RNA were removed by incubation with 1 μl each of DNAse I (1 U/μl) and RNAse A (20 mg/ml) in a final concentration of 1x DNAse reaction buffer (Invitrogen) for 15 min at room temperature. Enzymes were inactivated by addition of 1 μL of 25 mM ETDA and incubation at 65°C for 10 min. DNA was then extracted in multiple 50 μL aliquots using the Gentra Puregene Blood kit (Qiagen) per the manufacturer's recommendations. Samples were subjected to Proteinase K treatment and repeated protein precipitation steps as advised by the manufacturer. Final DNA extracts were rehydrated in 10 μL of sterile DNAse- and RNAse-free water (Gibco) at 4°C overnight to elute DNA pellets. The 10 μL DNA extracts from each of the 50 μl VC aliquots were pooled, and the isopropanol and ethanol precipitation steps were repeated to further concentrate DNA. The final DNA sample was again eluted in sterile water and stored at −20 °C.

Fosmid cloning was performed using the CopyControl Fosmid Library Production Kit (Epicentre) according to manufacturer's protocols with the following modifications. End-repaired DNA was immediately ligated without further size selection to avoid loss of material. Ligation of DNA into the CopyControl vector occurred overnight at 16°C. Twelve to twenty colonies were picked for each seasonal VC mix and grown overnight in selective media (LB + 12.5 μg mL^−1^ chloramphenicol) with addition of CopyControl Fosmid Autoinduction Solution at 1X final concentration to induce high copy number production of the fosmid vector. Fosmid DNA was purified from the high copy number induced overnight cultures using the FosmidMax DNA Purification Kit (Epicenter) and stored at −20 °C. Glycerol stocks of overnight cultures were stored at −80 °C.

To assess fosmid insert size and genetic differences, 2 μL of purified fosmid DNA was digested with Apa I at 25°C for 2 h followed by inactivation at 65°C for 20 min and visualized by pulsed-field gel electrophoresis (1% low melting point agarose gel, 0.5x TBE, 14°C, voltage gradient 6.0 V cm^−1^, total run time 22 h, initial switch time 1 s, final switch time 15 s, linear ramping factor). Each of six samples for sequencing (3 seasons × 2 depths) was composed of 2 μg of DNA from each of the 12 fosmids selected per sample based on differences in restriction digest patterns. Samples were sequenced using 454 Titanium chemistry (Cambridge, MA, USA).

#### Viral metagenomes

Viral DNA was extracted following the concentration of the 32 VCs by 30 kDa Centricon ultrafiltration into a single sample per depth (10 m and 200 m) and treatment to remove free DNA and RNA as stated above. Each sample was then divided in half for parallel DNA extraction. Two DNA extraction kits were used to minimize any potential bias in genomic extraction by either kit. Half the sample was extracted using Gentra Puregene Blood kit (Qiagen) as per the manufacturer's recommendations with the same modifications as listed for fosmid library DNA extraction. The second half of the sample was extracted using the QIAamp Virus MinElute Spin Kit (Qiagen) according to the manufacturer's protocol. Extracted DNA was frozen at −20°C until further processing. DNA from both protocols was thawed and combined just prior to multiple displacement amplification with random primers, per manufacturer's instructions (GenomePlex Complete Whole Genome Amplification kit, Sigma-Aldrich Canada Co, Oakville, Ontario, Canada), to increase DNA amounts prior to library construction. Amplified DNA was pooled within each depth to reflect a composite community to minimize seasonal biases.

For library construction, DNA was sheared by ultrasonication (Covaris M220 series, Woburn, MA) to approximately 250–300 bp. Sheared fragments were end-repaired, A-tailed and ligated to custom TruSeq adapters (IDT, Coralville, Iowa) using the NxSeq DNA Sample Prep Kit 2 for Illumina (Lucigen, Middleton, WI). After ligation of custom TruSeq adapters, an added heat-kill step (65°C for 20 min) was used to stop ligation; then, small fragments and adapter dimers were removed twice using Agencourt AMPureXP SPRI magnetic beads (Beckman Coulter, Danvers, MA). Libraries were checked for size and adapter dimers using a High Sensitivity DNA chip on a Bioanalyzer 2100 (Agilent) and quantified using Qubit (Invitrogen, Carlsbad, CA) according to the manufacturer's instructions. Libraries were sequenced for 2 × 250 bp paired-end reads on an Illumina MiSeq v2.0 at the Génome Québec Innovation Centre at McGill University (Montréal, Québec, Canada).

### Sequence analysis

#### Viral metagenomic data

Metagenomic sequences were trimmed for low quality base pairs and any residual adapter sequences using the default settings for Trimmomatic v0.30 (Bolger et al., 2014). All phiX reads from the control library were removed by mapping reads to the reference genome using the bowtie2 plugin (Langmead and Salzberg, 2012) in Geneious v7.1 (created by Biomatters and available from http://www.geneious.com/). All unassembled, paired reads were merged with FLASH using default minimum overlap settings (Magoč and Salzberg, 2011). The final “reads” dataset included all merged paired-end reads and all forward reads greater than 200 bp from the remaining non-overlapping sequence pairs. The unpaired reverse reads tended to be of poorer quality and were omitted from further analysis to avoid overestimation of sequence diversity. Sequence reads were annotated by BLASTx comparison to the complete viral protein RefSeq database (release 66, as of 10 July 2014) using MetaVir (Roux et al., 2011, 2014b). The taxonomic assignments and estimated community compositions were determined using the “Genome relative Abundance and Average Size GAAS” software package (Angly et al., 2009) implemented within MetaVir, which normalizes the distribution results based on reference viral genome length and weighs the similarity significance across multiple BLAST hits. The GAAS-derived community compositions and BLASTx differences with a bitscore cutoff of 50 were used for cross-sample comparisons. Sequences are available under the project “Saanich Inlet” and sample reads are designated as Saanich_10m_r200 (SI.10_m_) and Saanich_200m_r200 (SI.200_m_).

Rarefaction curves were compared by subsampling 50,000 sequences and determining sequence clusters at three nucleotide sequence similarity cutoffs (75, 90, 95%) in MetaVir. A dendrogram was calculated from BLAST-based comparison and clustered by overall similarity between Saanich Inlet and other publicly available viral metagenomes. Only metagenomes with more than 50000 sequences available in MetaVir were included in the cluster analysis (pvclust, R package; MetaVir).

#### Viral fosmid libraries

Fosmid sequences were assembled using the GS *De novo* Assembler (Newbler v2.5, Roche 454 Life Sciences) with default parameters. Vector sequences were trimmed and host *E. coli* sequences were screened for and removed from assemblies using GS *De novo* Assembler. Thirty-four fosmids larger than 30 kb were retained for further annotation and analysis, including six fosmids (SI.Prokaryotic) identified as virus-like sequences from a prior fosmid library from Saanich Inlet (Walsh et al., 2009). ORFs were called using Glimmer and Genemark plugins for Geneious v5.6, allowing for a minimum length of 150 bp and overlapping ORFs on either strand. ORFs were translated and annotated by searching against NCBI's “nr” database, as of 20 July 2014, by BLASTp with a minimum *e*-value of 10^−5^. Fosmid ORF annotations were verified by comparison against results from RAST (Aziz et al., 2008; Overbeek et al., 2014) and ACLAME (Leplae et al., 2009) by BLASTp searches using default settings and a minimum *e*-value of 10^−5^. Fosmids were also annotated as a contig project using MetaVir (project: Saanich Inlet; sample: Saanich_fosmids) by querying against the viral RefSeq database (release 66, 10 July 2014). Fosmids were aligned with specific viral reference genomes using the progressiveMAUVE plugin for Geneious v7.1 to determine sequence homology across an entire genome or fosmid sequence (Darling et al., 2010). Fosmid sequences were deposited in Genbank (KR029577-KR029610) and to CAMERA under the Moore Marine Phage/Virus Metagenomes as CAM_SMPL_000964 (Oxic_3), CAM_SMPL_000965 (Anoxic_3), CAM_SMPL_000971 (Anoxic_1); CAM_SMPL_000982 (Oxic_2), CAM_SMPL_000989 (Anoxic_2), and CAM_SMPL_000993 (Oxic_1).

#### Comparative fosmid, metagenomic, and single-cell genome sequence analysis

Metagenomic reads from Saanich Inlet (SI.10_m_, SI.200_m_) and Line P [from the Pacific Ocean Virome (POV) dataset (Hurwitz and Sullivan, 2013)] were queried by BLASTn against a custom database containing all viral genomes from RefSeq (release 66) and viral fosmid sequences from the Mediterranean Sea (Mizuno et al., 2013b) and Saanich Inlet (this study). For this analysis, only hits with a maximum *e*-value of 10^−5^, greater than 50 bp in alignment length, and greater than 90% nucleotide identity were considered significant to minimize potential error. Line P viral metagenomes, specifically, were queried to determine presence and relative abundance of SI fosmids in waters with similar environmental characteristics (Wright et al., 2012). Additional viral metagenomes from CAMERA (Table S2) were queried against Saanich Inlet fosmids by BLASTn using an *e*-value cutoff of 10^−5^. Long reference sequences provide more template and opportunity for read recruitment than short ones. As multiple reference genomes of varying length were included in RefSeq, and many are significantly larger than the average fosmid length from our study and in the Mediterranean Sea project (the databases under comparison), read recruitment to the reference databases were normalized according to reference genome size (per kbp) and metagenome size (per Gbp).

Regions of genetic similarity between the fosmid Oxic1_7 and the reverse complement of the putative archaeal provirus Pro_Nvie1 were determined by aligning both sequences with tBLASTx. Regions with an *e*-value less than 10^−5^ were plotted with genoPlotR in R (Guy et al., 2010) and included ORF annotations when available.

SI.10_m_ and SI.200_m_ sequences were recruited individually against viral fosmids (this study) and selected single-cell genomes (SAG) by bowtie2 using local recruitment and “high-sensitivity” in Geneious v7.1 resulting in recruitment of only reads at greater than 90% nucleotide identity. In brief, the SAG datasets used here originated from whole genome amplification and sequencing of single cells. These datasets were selected for their relevance to our study location [i.e., SAGs from the same location (Roux et al., 2014a)] and potential for discovery of novel viruses [marine thaumarchaea (Swan et al., 2014), and the Microbial Dark Matter project (Rinke et al., 2013)].

## Results

The genetic diversity of viral communities in the oxic and anoxic waters of Saanich Inlet was assessed through viral metagenomic data (Table S1) and large-insert fosmids (Table S3). Each fosmid represents a partial genome as it originated from a single strand of viral DNA. Overall, the fosmid sequences lacked similarity to known viral genomes as 5010 of 6459 (77.5%) ORFs across all 34 viral fosmids had no significant homology to viral reference genomes. However, annotation with NCBI's non-redundant database (nr) led to the identification of a putative virus infecting marine thaumarchaea. The viral community composition and genetic content in Saanich Inlet differed between the oxic and anoxic metagenomes from the viral size fraction (<0.22 μm) at 10 m (SI.10_m_) and 200 m (SI.200_m_), respectively. Collecting viral fosmid and metagenomic sequences from the same samples facilitated direct comparisons of the relative abundance of individual viral types through fragment recruitment of metagenomic reads to identify major contributors to the Saanich Inlet viral assemblages. Additionally, detailed fragment recruitment of viral metagenomic sequences to single-cell genomes (SAGs) revealed prokaryotic genomic regions that are likely from viruses that infect marine thaumarchaea and proteobacteria in the SUP05 clade. Details of these results are presented below.

### Saanich inlet viral communities are primarily comprised of viruses with no homology to other virus isolate genomes

#### Diversity within viral metagenomes

**Figure 1:**
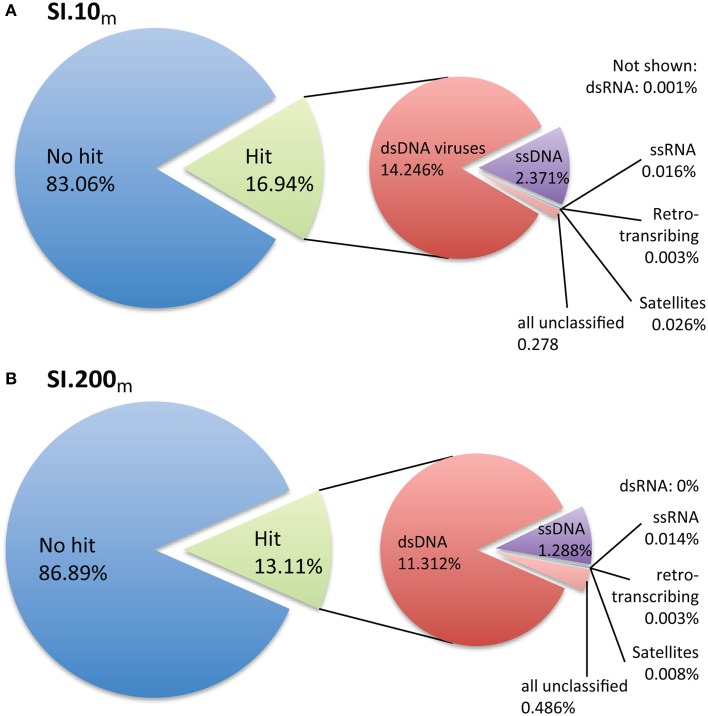
**Taxonomic assignment of metagenomic reads based on viral reference genomes**. **(A)** SI.10_m_ sequences are from oxic waters at 10 m (*n* = 2.052,047) and **(B)** SI.200_m_ sequences are from waters that are generally anoxic at 200 m (*n* = 1.728,968). Percentages listed are the percent of sequences classified from all metagenomic data per sample.

Only 16.9% (SI.10_m_) and 13.1% (SI.200_m_) of the sequences could be taxonomically assigned based on significant BLASTx hits to the non-redundant viral genomes in RefSeq (Figure 1). The sequences with taxonomic hits were primarily dsDNA viruses from the Order *Caudovirales* (Figures S1, S2) based on GAAS-computed community composition estimates that account for genome length variation among viral taxa (Angly et al., 2009). Within the dsDNA virus fraction, podovirus-like reads were the most abundant (SI.10_m_: 34.7%, SI.200_m_: 38.9%), while slightly fewer reads were assigned to siphoviruses (SI.10_m_: 28.5%, SI.200_m_: 36.4%); other viruses were 17.7% (SI.10_m_) and 15.4% (SI.200_m_), while myovirus-like reads comprised 9% (both SI.10_m_ and SI.200_m_) and unclassified viruses in the *Caudovirales*, 5% (SI.10_m_) and 4% (SI.200_m_). Viral taxa that each recruited more than 5% of the dsDNA virus reads included *Persicivirga* phage P12024L (SI.10_m_), *Peligibacter* phage HTVC010P (SI.10_m_) and *Vibrio* phage pYD21-A (SI.200_m_). Other dsDNA viruses that each recovered ~2% of the reads in SI.10_m_ included *Roseobacter* phage SIO1, *Peligibacter* phage HTVC011P, *Peligibacter* phage HTVC019P, *Celeribacter* phage P12053L, and *Cellulophaga* phage phi10:1 (Figure S1). In SI.200_m_, ~2% of the dsDNA reads were assigned to *Peligibacter* phage HTVC010P, *Puniceispirillum* phage HMO-2011, and phages of *Cellulophaga* and *Vibrio* spp (Figure S2). Sequences assigned to phycodnaviruses accounted for only 0.38% (SI.10_m_) and 0.26% (SI.200_m_) of the dsDNA reads, and ssDNA viruses totaled 14% (SI.10_m_) and 10% (SI.200_m_) of all classified metagenomic sequences.

The estimated richness and overall sequence similarity in the Saanich Inlet viral metagenomes were similar to others from the open ocean, but were higher in SI.10_m_ than SI.200_m_ across three nucleotide similarity cutoffs of 75, 90, and 98% when looking at all sequence reads (Figure S3) and when metagenomes were sub-sampled to 50,000 reads (Figure S4). Neither metagenome was sequenced to completion given that the rarefaction curves remained near linear after accounting for all sequences (Figure S3). Re-sampling 50,000 reads per viral metagenome facilitated comparisons across many viral metagenomes obtained with different sequencing efforts. All available marine viral metagenomes were also under-sampled given that ~48,000 sequence clusters were formed on average per 50,000 sub-sampled reads (Figure S4). At 50,000 sampled reads per viral metagenome and 90% nucleotide similarity, SI.10_m_ had 48,806 and SI.200_m_ had 45,980 clusters, and yielded a similar number of sequence clusters as for data from other oceanic viral metagenomes (Figure S4).

Depth and region-based clustering of viral metagenomic data was observed by searching for sequence similarity, despite the effects from under-sampling (Figure S5). Two major clusters were resolved using sequence similarity rather than taxonomic community compositions. One cluster included only oxic viral metagenomes, while the second was comprised of viral metagenomes from below the chlorophyll maximum or from anoxic or low oxygen zones of the water column. The surface or oxic cluster also contained sub-clusters by geographic region (i.e., Line P in the northeast subarctic Pacific Ocean, Line 67 off the coast of Monterey CA, Scripps Pier in San Diego CA, etc…). SI.10_m_ grouped with other surface ocean viral metagenomes, but not within any of the regional clusters. The low-oxygen or anoxic viral metagenomes were less structured by region than the surface ocean or oxic metagenomes. SI.200_m_ fell outside of either cluster, making it distinct from viral metagenomes from across the Pacific Ocean. A third major cluster included viral metagenomes from samples pooled from several depths [Arctic_Vir, Gulf_of_Mexico, and British_Columbia, (Angly et al., 2006)], viral metagenomes with shorter sequence lengths (≤250 bp), and one viral metagenome with notable non-marine inputs (Coral_Atoll_Kiritimati, Dinsdale et al., 2008). Although read length may have contributed to the clustering pattern, some of the viral metagenomes with shorter read lengths (<250 bp) also appeared in the surface-oxic cluster. In general, viral communities from the surface ocean were distinguishable from those at depth or with lower oxygen concentrations.

#### Diversity of viral fosmids

**Figure 2:**
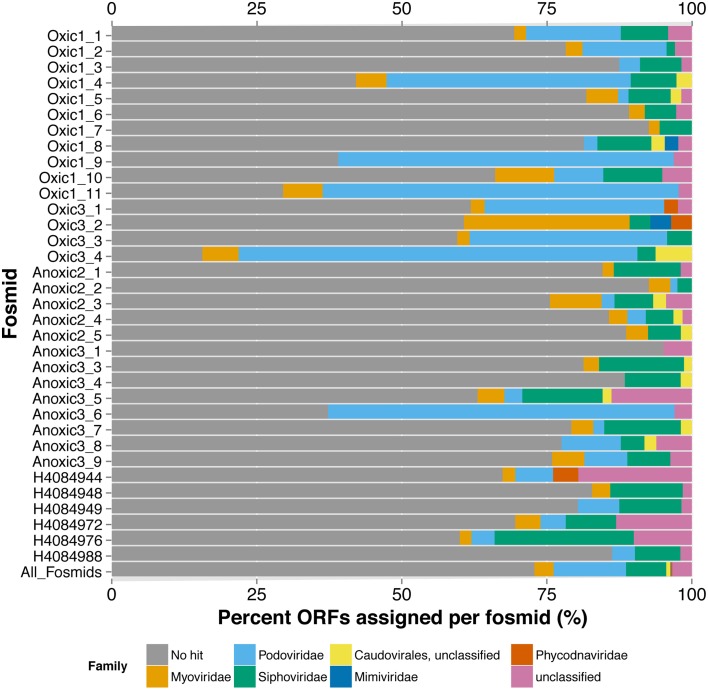
**Taxonomic Annotation of Saanich Inlet Fosmids**. Classifications are shown per fosmid (each bar) by percent of ORFs assigned to each viral family (x-axis). Bars are colored by classification at family level.

From the few recognizable sequences, the taxonomic assignment or classification of each fosmid can offer insights into the virus' lifestyle and possible hosts. Similar to the metagenomes, the limited number of ORFs in the viral fosmids with homology to a viral genome in the RefSeq dataset were primarily similar to members of the Order *Caudovirales* by the best BLASTx hit of each ORF and the last common affiliation (consensus) of all BLASTx hits recovered per fosmid (Figure 2, Table S3). The fosmids had significant sequence similarity to several known marine viruses, including pelagiphages, cyanophages, and phages of *Celluphaga* and *Puniceisprillum*. Only five of 34 fosmids, Oxic1_4, Oxic1_9, Oxic1_11, Oxic3_4, and Anoxic3_6, had more than 50% of its ORFs annotated by BLASTx similarity to a protein previously recovered from a viral genome. Three of these fosmids, Oxic1_9, Oxic1_11, and Anoxic3_6, had several ORFs in common with the *Pelagibacter* (SAR11) phage HTVC010P (Figure S6), with Anoxic3_6 and Oxic 1_9 being most similar (52.3% pairwise nucleotide identity) despite an unaligned gap near the putative tail fiber ORFs. Fosmid Oxic1_4 was most similar to another *Pelagibacter* phage HTVC011P and Oxic3_4 was found similar to several *Synechococcus* phage genomes (Syn5, P60, S-CBP42, S-SSM4). Other notable assignments included viruses infecting the genera *Rhizobium, Streptococcus, Vibrio, Enterobacteria* and *Dunaliella*, although confidence in these assignments was limited due to the lack of consistent taxonomy within a fosmid and low amino-acid similarities. Gene assignments by MetaVir were consistent by gene name with manual annotations by BLASTx to the non-redundant database “nr”; taxonomic affiliations from “nr,” however, skewed toward prophage regions in cellular organisms and viral fosmid sequences from the Mediterranean Sea (Mizuno et al., 2013b). In summary, annotation of the Saanich Inlet fosmid sequences against reference viral genomes indicated the presence of pelagiphage-like viruses, cyanophages, and many unassigned viruses.

### Fragment recruitment of metagenomic reads indicates distribution by depth

**Figure 3:**
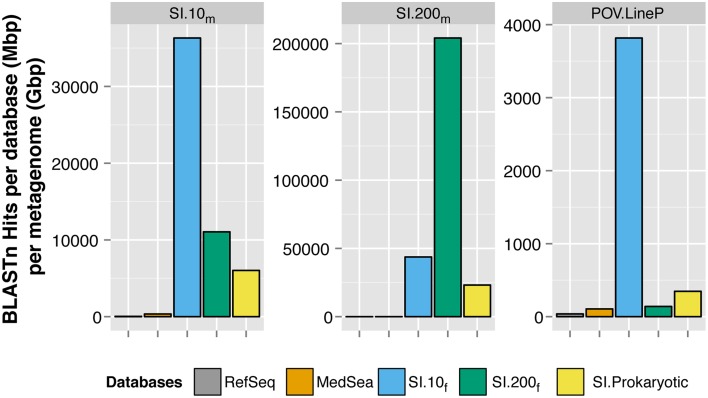
**Recruitment of metagenomic sequences to viral reference genomes and fosmids**. Each panel represents a BLASTn search of reads from SI.10_m_ (left), SI.200_m_ (center), and LineP viromes (POV.LineP) against viral genomes in RefSeq, Mediterranean Sea (MedSea) viral fosmids and Saanich Inlet viral fosmids (SI.10_f_, SI.200_f_, SI.Prokaryotic). The cumulative number of hits returned per total bp in each fosmid or genome collection (Mbp) per gigabasepairs (Gbp) of metagenomic sequence data (y-axis) is shown. Note y-axis is different for each panel.

Saanich Inlet fosmids recruited more viral metagenomic sequences than the viral genomes in RefSeq or viral fosmids from the Mediterranean Sea (Figure 3). Only 0.17% (SI.10_m_) and 0.05% (SI.200_m_) of the metagenomic reads had significant sequence similarity to any of the 5580 viral genomes in RefSeq, using a more stringent cutoff of 90% nucleotide identity, maximum *e*-value of 10^−5^ and minimum alignment length of 50 bp. Mediterranean Sea viral fosmids (Mizuno et al., 2013a,b) recruited 0.25 and 0.04% of sequences from SI.10_m_ and SI.200_m_, respectively. Lastly, all Saanich Inlet viral fosmids collectively recruited 0.78 and 3.78% of sequences from SI.10_m_ and SI.200_m_, respectively. Fosmids from 10 m (SI.10_f_) recruited 0.6% of reads from SI.10_m_ while fosmids from 200 m (SI.200_f_) recruited only 0.15% from SI.10_m_. Conversely, SI.200_f_ recruited more reads from SI.200_m_ (2.89%) than SI.10_m_ (0.75%). Viral fosmids identified from the cellular fraction (SI.Prokaryotic) recruited an additional 0.03% of reads from SI.10_m_ and 0.14% of reads from SI.200_m_. SI.10_f_ also recruited the most reads from the Pacific Ocean Virome Line P (POV.LineP) viral metagenomes, which included viral metagenomes from both surface and deep waters.

**Figure 4:**
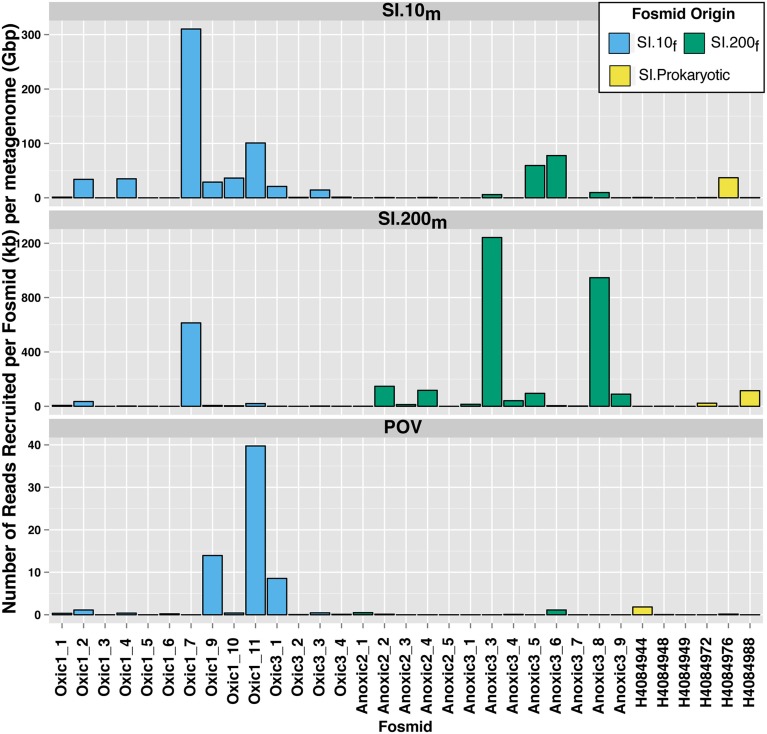
**Differential recruitment of metagenomic reads to Saanich Inlet fosmids**. The number of hits per fosmid was normalized as the number of BLASTn hits per kb of fosmid length per gigabasepairs of metagenomic sequence data (y-axis).

Read recruitment from the Saanich Inlet viral metagenomes was unevenly distributed among fosmids (Figure 4). Four fosmids (2 from SI.10_f_, 2 from SI.200_f_) recruited more than 50 reads per kb (fosmid length) per Gbp (metagenome) from SI.10_m_. Eight fosmids (1 from SI.10_f_, 6 from SI.200_f_, 1 from SI.Prokaryotic) recruited more than 50 reads per kb (fosmid length) per Gbp (metagenome) from SI.200_m_. Additionally, the four fosmids that recruited the most reads from POV.LineP were all pelagiphage-like (Figure 4) although the number of reads recruited by each fosmid differed. Anoxic3_6 and Oxic1_11 recruited over 1500 reads from SI.10_m_ (77.7 and 100.9 reads per kb per Gbp, respectively) compared to 89 reads and 307 reads (5.1 and 20.6 reads per kb per Gbp, respectively) from SI.200_m_. In contrast, Oxic1_9 recruited 579 and 112 reads from SI.10_m_and SI.200_m_ for 28.8 and 6.3 reads per kb per Gbp, respectively. The fosmids from 10 m, in general, recruited more reads from SI.10_m_ than SI.200_m_ and fosmids from 200 m recruited more reads from SI.200_m_ than SI.10_m_.

When compared to viral metagenomic data from many different sources, Saanich Inlet fosmids were more similar to viral sequences from marine rather than non-marine sampling locations (Table S2). Specifically, the fosmids primarily recruited sequences from samples in the Moore Marine Phage/Virus Metagenomes project (CAM_PROJ_BroadPhage), which contains viral metagenomic data from throughout the world's oceans. The fosmids with the most metagenomic hits across all of these metagenomes were Oxic1_6, Oxic1_8, Anoxic2_1, Oxic1_1, and Anoxic2_3. Three pelagiphage-like fosmids, Oxic1_9, Oxic1_10, Oxic1_11, recruited reads from nine different metagenome projects, indicating these fosmid sequences originated from viruses widespread in the environment.

### Virus discovery by paired analysis of “omic” datasets

#### Leveraging the non-redundant “nr” NCBI database uncovered genomic evidence for putative marine thaumarchaeal viruses

**Figure 5:**
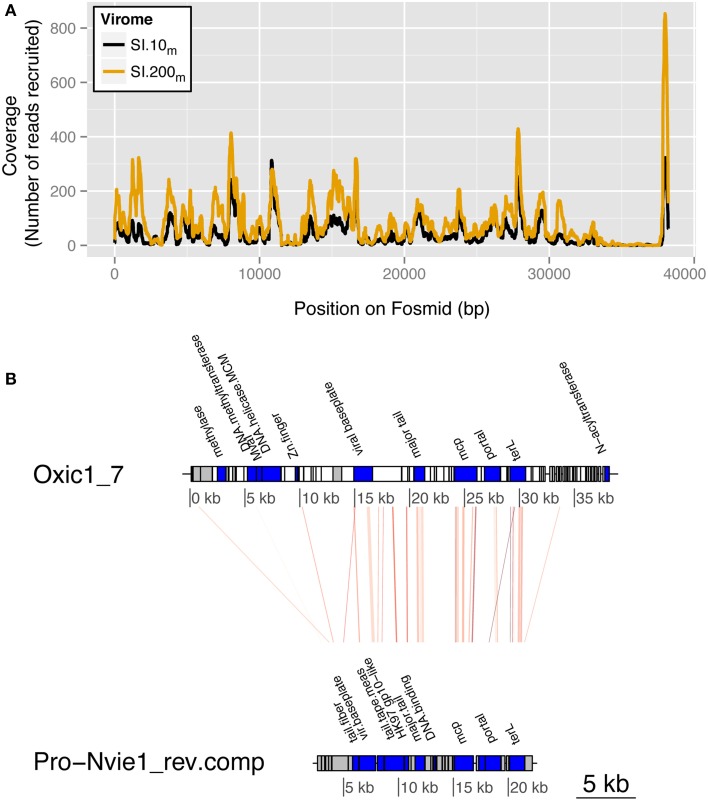
**Saanich Inlet fosmid Oxic 1_7 is a putative thaumarchaeal virus**. **(A)** Fragment recruitment coverage (y-axis) by position (bp) along the fosmid (x-axis) is shown for SI.10_m_ (black) and SI.200_m_ (gold) for reads with greater than 90% nucleotide identity. **(B)** Regions of genetic similarity by tBLASTx hits to thaumarchaeal provirus Nvie1 (reverse complement) with an *e*-value cutoff of 10^−5^. The red lines connect the regions of genetic similarity as determined by tBLASTx between the fosmid Oxic1_7 and the provirus Nvie1. ORF blocks are shaded to indicate annotated genes (blue), hypothetical proteins (gray), or no hit in a database (white).

Manual annotation of the fosmids against the non-redundant “nr” reference database (NCBI) revealed a putative host for fosmid Oxic1_7, the most well-represented fosmid in both Saanich Inlet viral metagenomes. Viral metagenomic reads were recruited across all of Oxic 1_7 except between 35 and 38 kb (7467 reads from SI.10_m_ and 13,696 reads from SI.200_m_; Figure 5A). Four of 193 ORFs had sequence similarity to siphoviruses, including one hit to the archaeal BJ1 virus when querying the viral reference genomes alone (MetaVir). When the fosmid ORFs were queried against “nr,” 25 of 193 ORFs had significant hits (Figure 5B). Seven of these ORFs matched the putative thaumarchaeal provirus, Pro-Nvie1, that occurs in the genome of the ammonia-oxidizing thaumarchaeon Candidatus *Nitrososphaera viennensis* strain EN76 isolated from soil (Krupovic et al., 2011). These ORFs included hallmark viral sequences that putatively encode for: terL (terminase, large subunit), protease/major capsid proteins, and tail proteins with an average 30% amino acid identity. Other Oxic1_7 ORFs were similar to DNA methylases and helicases found in other archaea (average 53% amino acid identity across four ORFs) and bacteria (average 50% across 11 ORFs to *Firmicutes*). The three remaining ORFs were similar to hypothetical proteins found in *Batrachochytrium dendrobatidis* (*n* = 1) and EBPR siphovirus 2 (*n* = 2).

#### Identifying regions of possible viral origin within single-cell genomes

**Figure 6:**
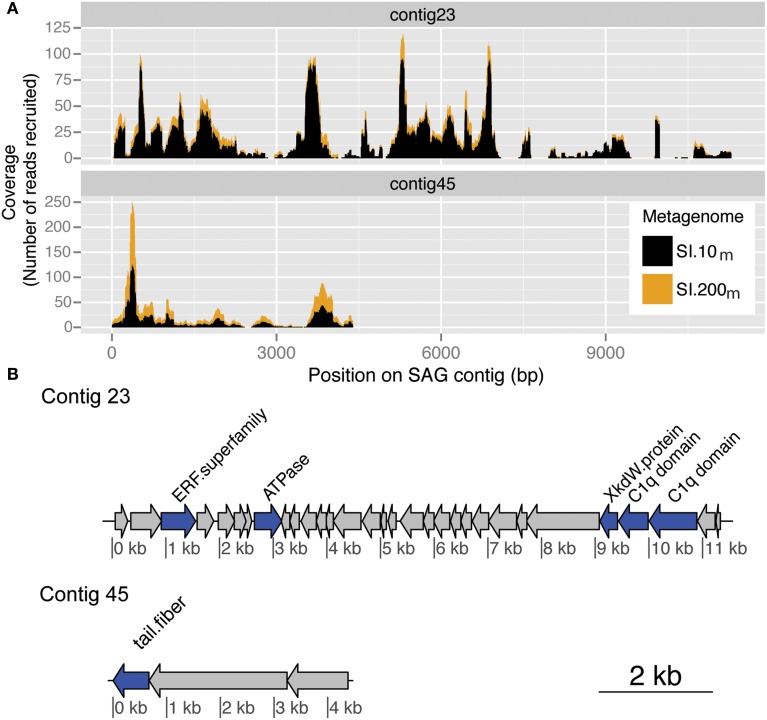
**Putative viral contigs recovered from a marine thaumarchaeal SAG**. **(A)** Coverage by metagenomic reads from SI.10_m_ (black) and SI.200_m_ (gold) with greater than 90% nucleotide similarity to a marine thaumarchaeal SAG for contig 23 and contig 45. Coverage is cumulative (stacked height of histogram) across both metagenomes. **(B)** ORF annotations for each SAG contig as described in the Integrated Microbial Genomes (IMG) system. ORF color indicates whether it is a named (blue) or hypothetical (gray) protein.

Putative viral regions in thaumarchaeal and SUP05 SAGs were identified and confirmed by recruitment of viral metagenomic sequences to contigs within the SAGs (Figure 6). The two examples detailed below are for host organisms for which little is known about possible host-virus interactions in the ocean. SI.10_m_ and SI.200_m_ were also recruited against the “Microbial Dark Matter” SAGs from Rinke et al. (2013), but no single contig recruited a significant number of sequences that were distributed somewhat evenly across a region equivalent to a few viral genes.

By recruiting viral metagenomic reads, putative viral regions on contigs reported from SUP05 SAGs were independently verified, particularly with sequences from SI.200_m_. The same viral contigs and regions were identified as putative viral sequences from marine bacteria SUP05 through the presence of hallmark viral genes (Roux et al., 2014a). Two SUP05 SAGs, in particular, recruited up to 15,643 reads per contig from SI.200_m_. The first SUP05 SAG (AB.754.J03AB.906) recruited 22,369 reads from SI.200_m_ and 303 reads from SI.10_m_ to 6 of its 43 contigs. A second SAG (AB750C22AB.904) recruited 6894 sequences from SI.200_m_ and 71 reads from SI.10_m_ to 7 of its 63 contigs. The highest recruiting contig from a SUP05 SAG (AAA160.G15) from SI.10_m_ recovered only 831 reads, which was not surprising given that SUP05 is typically observed in anoxic waters (Walsh et al., 2009; Wright et al., 2012). This analysis confirmed the presence of these viruses or their close relatives as members of the viral assemblage (or organisms smaller than 0.2 um size fraction) in the anoxic zone of Saanich Inlet.

The same approach was followed to identify putative viral regions in marine thaumarchaea SAGs (Figure 6). One thaumarchaeal SAG (AAA288-I14) from Station ALOHA recruited metagenomic reads from both 10 m and 200 m in Saanich Inlet across contigs 23 and 45. Contig 23 recruited 1199 reads from SI.10_m_ and 246 reads from SI.200_m_(total = 1445 reads) and contig 45 recruited 371 reads from SI.10_m_ and 63 reads from SI.200_m_ (total = 444 reads). Average coverage was 15.4-fold (SI.10_m_) and 3.4-fold (SI.200_m_) for contig 23 and 13.5-fold (SI.10_m_) and 2.6-fold (SI.200_m_) for contig 45. The two SAG contigs included ORFs which encode for a putative phage tail fiber and other hypothetical proteins found in marine phage genomes (Swan et al., 2014). Viral metagenomic read recruitment to an additional 26 archaeal SAGs from Saanich Inlet resulted in recruitment of 10 or fewer reads each. Thus, these additional SAGs either did not encode genetic content similar to sequences captured in the viral metagenomes or lacked viral regions altogether due to incomplete genome sequencing or natural variation.

## Discussion

Advances in nucleic-acid technologies have led to huge increases in viral sequence data; yet, most of these environmental sequences are orphans without a genomic context. Finding a genomic home for these data and ultimately elucidating a function for this viral “dark matter” requires representative virus reference genomes, which can be used to recruit viral metagenomic data. Viral reference genomes may originate from cultured isolates, but with few exceptions, the lack of representative host strains in culture and the enormous microbial diversity in nature means that it is untenable to bring most of the representative cellular diversity into culture. Thus, the vast majority of marine viral reference genomes will not be acquired using culture-based approaches. Moreover, the vast viral sequence diversity in aquatic systems and the relatively short reads provided by current high-throughput sequencing technologies makes it intractable to confidently assemble complete genomes from metagenomes except for RNA (e.g., Culley et al., 2006, 2014) and ssDNA viruses (Tucker et al., 2010; Labonté and Suttle, 2013a,b), which have very small genomes. For dsDNA viruses, reference genomes need to be derived from sequencing large fragments of viral DNA, such as are captured by fosmid cloning (e.g., Garcia-Heredia et al., 2012), targeted metagenomics (e.g., Martinez-Martinez et al., 2014), or potentially single-virus genomes (Allen et al., 2011). Viral reference genomes can provide templates against which metagenomic data can be recruited and placed in a genomic context, which will facilitate closing some of the gaps in our knowledge regarding marine virus genomes; these are both essential steps forward to understanding the ecology of marine viruses (Culley, 2013; Bibby, 2014). Our study contributes 34 new partial viral reference genomes from fosmid cloning and sequencing, and identifies genomic fragments of putative viruses infecting marine thaumarchaea and SUP05 proteobacteria. This study also demonstrates that relying on annotation using genomes from cultured viruses alone is a barrier to the discovery of new virus taxa. Moreover, the repeatable recovery of highly similar sequences from both the virus and cellular size fractions of seawater indicates that these sequences likely represent active and common viruses within the marine environment that should be targeted for further investigation.

Combining nucleic-acid sequencing technologies (metagenomic, fosmid, and single-cell genomic datasets) to explore viral diversity and virus-host interactions allowed orphaned metagenomic data from Saanich Inlet to be placed into a genomic context and showed that Saanich Inlet viral communities are distinguishable from those in other environments. Recruitment of metagenomic reads to SAGs highlighted genomic islands of likely viral origin. Comparative analyses between viral fosmid sequences and SAGs uncovered previously unknown viruses and host-virus relationships, such as the putative thaumarchaeal virus sequence from fosmid Oxic1_7. In particular, data from the oxygen-minimum zone provided strong evidence for the presence of these putative viruses infecting marine thaumarchaea and SUP05 proteobacteria, emphasizing that viruses in these environments are relatively understudied. These results show the power of combining environmental genomic approaches to illuminate viral “dark matter” and are discussed in detail below.

### Taxonomic identification of viral metagenomic sequences in saanich inlet was limited

Prokaryotic communities differ between anoxic waters found at depth and oxic surface waters; thus viral communities would also be expected to differ (e.g., Cassman et al., 2012). However, the scenario in Saanich Inlet is more complex, as stratification is perturbed by deep water renewal shoaling anoxic/sulfidic bottom waters upwards with concomitant changes in microbial community composition (Zaikova et al., 2010; Wright et al., 2012). As samples from the same depth were pooled across time, this may be one reason that Saanich Inlet virus communities were not as easily distinguished from those at other marine locations when looking at family- and order-level taxonomic classifications. Metagenomic sequences were assigned to many taxa of marine viruses when queried against RefSeq, a database of viral reference genomes (Figure 1, Figures S1, S2). As this reference database is dominated by sequences from viruses within the *Caudovirales*, this in turn dictated the taxonomic placement of the metagenomic reads. For both metagenomes, ~85% of the metagenomic reads were not assigned to a taxonomy (Figure 1). In fact, the percent of metagenomic reads classified per sample ranged from 0.1 to 28.5% (average 15.2%) across all marine viral metagenomic datasets (e.g., Line P, Line 67, Scripps Pier) used for comparative analyses. Although SI metagenomic samples were not sequenced to completion, the diversity estimates were similar to those obtained from other ocean sites (Figure S4). What is evident from the data is that improved classification and ecological interpretation of viral metagenomic data requires more representative viral genomes in the reference databases.

Comparisons based on taxonomic classification may obscure differences among samples as most sequences remain unassigned, but clustering by sequence similarity resolved differences in viral metagenomic data from oxic and anoxic waters (Figure S5). Saanich Inlet sequences grouped with POV metagenomic data according to depth, consistent with oxygen concentrations in the two environments; this is one of the few comparisons to demonstrate clustering of viral metagenomic data by ecological niche (Hurwitz et al., 2015). These results collectively suggest that the composition of viral communities is predictable based on abiotic or biotic influences in the local environment.

The taxonomic identities assigned to the metagenomic sequences also included many ssDNA viruses. Although several reference genomes from ssDNA viruses have been assembled from other Saanich Inlet metagenomic data (Labonté and Suttle, 2013a,b), the percent contribution of metagenomic sequences belonging to ssDNA viruses in this study is likely over-estimated due to the biases associated with multiple displacement amplification (Polson et al., 2010; Kim and Bae, 2011). For this reason, our analysis focused on dsDNA viral communities and the novel diversity recovered in the metagenomic data in relation to the viral fosmid and single-cell data from this study and others.

### Viral diversity recovered by fosmids as genome proxies

Fosmid cloning and sequencing has been used for recovering complete and partial viral genomes from seawater (Mizuno et al., 2013a,b)and hypersaline environments (Garcia-Heredia et al., 2012). Although it is low throughput and time-intensive, fosmid cloning captures up to ~40 kb of DNA from a single virus; whereas metagenomic sequences lack a genomic context. Both methods, however, yield viral genomic data without the need for culturing. Fosmid cloning may also facilitate recovery of uncommon taxa due to bias for sequences with higher G+C content (Danhorn et al., 2012).

Isolate-based viral genomes provided excellent templates against which closely related fosmids could be compared. As proof of principle, the genomes of pelagiphages, which are common in the marine environment and abundant in most viral metagenomic data (Zhao et al., 2013), were compared to three SI fosmids that had multiple BLASTx hits to pelagiphage ORFs (Figure S6). The comparisons confirmed that based on genomic content and organization the fosmids contained DNA from close relatives of pelagiphage isolates, although there was evidence of population or strain differences between the isolates and the viruses represented by the fosmids. Annotation of the remaining fosmids using traditional reference databases provided clues as to the taxonomic classification of each fosmid and their potential hosts (Figure 2, Table S3). However, these results were often inconclusive due to different taxonomic assignments by BLASTx similarity to ORFs within a single fosmid and so demonstrate the novelty of the representative viruses captured by the fosmid sequences.

### Virus ecology inferred from paired analysis of molecular datasets

More than 1000 fosmids covering a spectrum of viral taxa have been sequenced from the Mediterranean Sea (Mizuno et al., 2013a,b; Rodriguez-Valera et al., 2014), yet the 34 viral fosmids sequenced from Saanich Inlet in this study recruited more metagenomic sequences than all of the MedSea fosmids combined. Thus, a larger proportion of metagenomic sequences from Saanich Inlet could be assigned to a genomic context using locally derived fosmid sequences than could be assigned to a much larger database of fosmid sequences from another location (Figure 3). There was minimal overlap in the metagenomic sequences from each database that were recruited to the fosmids; the SI fosmids tended to have higher sequence similarity to the metagenomic sequences than did the MedSea fosmids for the few that did overlap (data not shown). Given the environmental differences between the locations and sampling depths, these results are not surprising but show that viral communities are specific to their environment, consistent with the metagenomic sequence similarity clustering patterns (Figure S5).

Recruitment of metagenomic sequences from SI.10_m_ and SI.200_m_ to the SI fosmids also highlighted depth-dependent distributions as fosmids from 10 m recruited more sequences from SI.10_m_ than SI.200_m_ and vice versa (Figures 3, 4). These differences persisted against a backdrop of sample pooling, DNA amplification during sample preparation, and seasonal dynamics that would be expected to mask these differences. In particular, Anoxic3_3 and Oxic 1_7 represent key members of the Saanich Inlet viral assemblage during our study. Several hundreds to thousands of metagenomic sequences shared similarity with these two fosmids that represent two distinct viral genomes (Figure 4). As such, they may be excellent targets for developing PCR primer sets that could be used to track fluctuations in virus populations over time and depth or to query single-cell genomes to identify possible hosts.

### Virus discovery through fosmids as genome proxies

Evidence for marine archaeal viruses can be inferred from the similarity of sequences recovered from the free virus size fraction (<0.22 μm) in Saanich Inlet to a putative thaumarchaeal provirus (this study), CRISPR regions in a thaumarchaeal genome (Spang et al., 2012) and in metagenomic data from a hypersaline lake (Emerson et al., 2013), and detection of genomic islands in single-cell genomes (Swan et al., 2014). Manual annotation with “nr” provided a consistent taxonomic assignment for fosmid Oxic1_7 as a plausible virus of marine thaumarchaea, an identity that would have been missed by annotation using viral reference genomes alone (Figure 5). The identification stems from significant sequence similarity to a putative provirus genome, Pro-Nvie1, recovered from a soil archaeal genome. Highly similar metagenomic sequences with greater than 90% nucleotide identity to Oxic1_7 were recovered from both sampled depths, confirming the presence of a closely related thaumarchaea virus in the viral size fraction at Saanich Inlet. The recovery of several ORFs within Oxic1_7 with similarity to the same provirus and the hyperthermophilic archaeal virus BJ1 implies that the DNA from this fosmid originated from an archaeal virus.

Viral sequences occurred in single-cell genomic data from bacteria belong to the SUP05 clade (Roux et al., 2014a), which are abundant in the anoxic waters of Saanich Inlet (Walsh et al., 2009), and in metagenomic data from seawater and vent fluid from the Lau Basin (Anantharaman et al., 2014). Highly similar sequences to SI.200_m_ were found in the SUP05 single-cell genomic data, but not in metagenomic data from Lau Basin, likely due to the many environmental differences between the sites. Many sequences from SI.200_m_ were also similar to contigs from thaumarchaeal SAGs from the Pacific Ocean (Swan et al., 2014), that also contained putative viral genes (Figure 6).

Marine thaumarchaeal viruses as active players in the ocean, particularly in OMZs, would directly affect biogeochemical cycling. The viral shunt is most often viewed in the context of viral-driven recycling of carbon, nitrogen, and phosphorus (Fuhrman, 1999; Wilhelm and Suttle, 1999; Shelford et al., 2012; Weitz and Wilhelm, 2012). In addition to being key nitrifiers in the ocean (Francis et al., 2007), marine thaumarchaea have also been implicated as important remineralizers of cobalamin (vitamin B12) in the ocean (Doxey et al., 2015). Many cellular processes require cobalamin as an enzymatic cofactor and it is often a limiting factor in cellular growth (Sañudo-Wilhelmy et al., 2014). If highly active, viral infection of marine thaumarchaea and SUP05 would have significant potential effects on the nitrogen, sulfur and other biogeochemical cycles that are unaccounted for in the current flux budgets.

## Concluding remarks

Viral ecology has benefited greatly from the adoption of nucleic-acid technologies to assess viral diversity and coding potential. Higher-throughput sequencing, lower costs, and new methods to recover, amplify, and target viral particles and nucleic acids are continuing to push research in new directions. Although assessment of the taxonomic composition of the Saanich Inlet viral community was limited by the availability of reference genomes, the 34 new fosmid sequences obtained in this study provided a genomic context for a significant and otherwise orphaned proportion of the viral metagenomic data. Taken together, these results highlight the power of combining sequencing approaches and the resulting data to interrogate viral diversity and discover potential virus-host interactions. For example, our analysis of viral metagenomic, fosmid sequences, and prokaryotic single-cell genomes together provided genetic evidence for a likely active and common presence of viruses infecting thaumarchaea in the global ocean. These findings may have been less convincing if each dataset was only considered on its own. Additionally, this study is one of only a handful of studies completed to date that demonstrates clustering of viral communities by ecological niche using metagenomic data. Strategic sampling and genetic exploration of under-explored areas, such as anoxic waters, will provide important resources for understanding not only the genetic diversity and genetic potential of marine viruses but also their contributions to nutrient cycling and ecosystem services.

## Author contributions

DMW and CAS conceived the study. DMW and RAW carried out the laboratory work and CTC, DMW, and RAW conducted the bioinformatics analyses. CTC and DMW wrote the paper with input and revisions from CAS. RAW and SJH contributed to discussions of the results and article content. All authors have reviewed and agreed to the final content.

### Conflict of interest statement

The authors declare that the research was conducted in the absence of any commercial or financial relationships that could be construed as a potential conflict of interest.
