# Supplementary material for: Combining genomic sequencing methods to explore viral diversity and reveal potential virus-host interactions
Source: Front Microbiol. 2015 Apr 10;6:265. doi: 10.3389/fmicb.2015.00265 (PMC4392320; doi:10.3389/fmicb.2015.00265)

Figure S2. Taxonomic GAAS-computed composition of viral metagenomic reads in Saanich Inlet for $SI.200_m$.

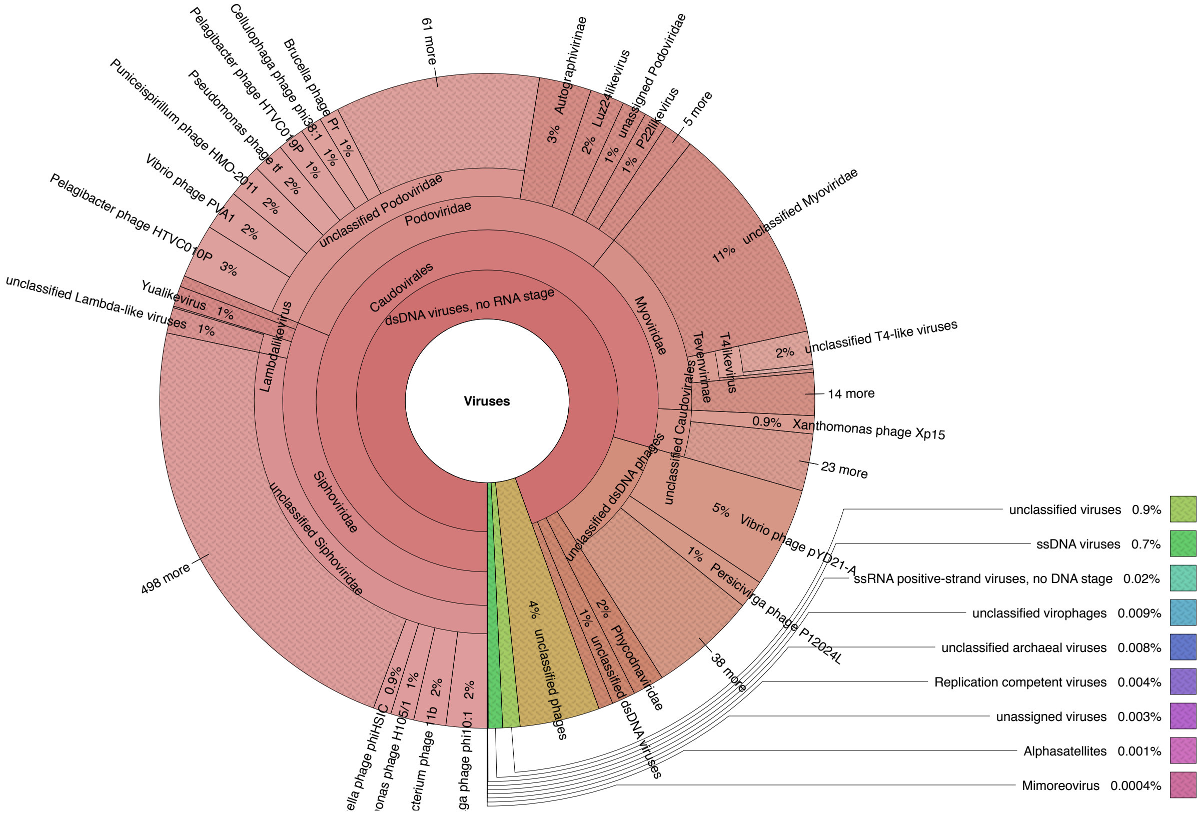

Supplement: Supplementary file 5 [file Image2.PDF]
