# Supplementary material for: Combining genomic sequencing methods to explore viral diversity and reveal potential virus-host interactions
Source: Front Microbiol. 2015 Apr 10;6:265. doi: 10.3389/fmicb.2015.00265 (PMC4392320; doi:10.3389/fmicb.2015.00265)

Figure S3. Rarefaction curves by sequence clustering at 90 and 98% sequence similarity for the each viral metagenome. The number of unique clusters is shown (y-axis) relative to the given number of sequences included (x-axis), using all sequences greater than 200bp in length.

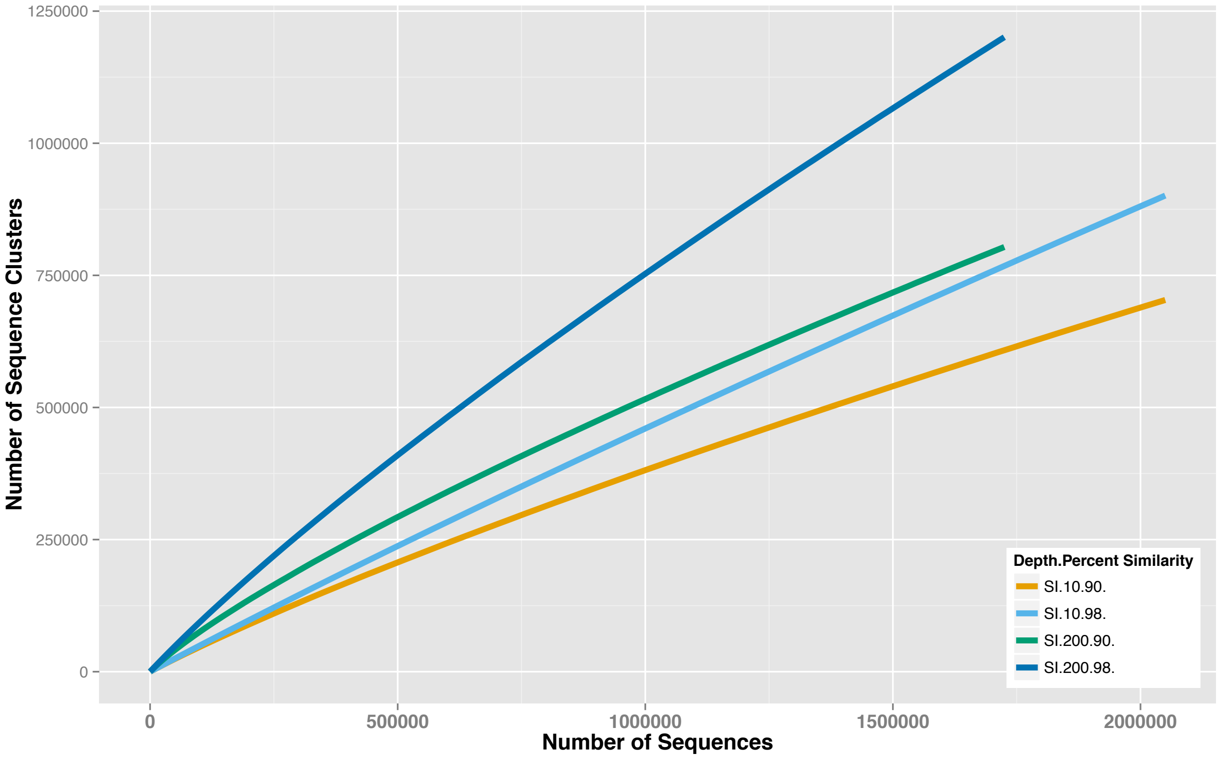

Supplement: Supplementary file 6 [file Image3.PDF]
