# Supplementary material for: Combining genomic sequencing methods to explore viral diversity and reveal potential virus-host interactions
Source: Front Microbiol. 2015 Apr 10;6:265. doi: 10.3389/fmicb.2015.00265 (PMC4392320; doi:10.3389/fmicb.2015.00265)

Figure S4. Rarefaction curves indicate similar levels of richness across several viral metagenomic datasets. (A) Comparison of rarefaction curves are based on sequence clustering at three nucleotide similarity levels (75%, 90%, 98%), re-sampled to 50,000 reads per metagenome. Comparison by sampling location **(B)** and water column depth **(C)** of the mean endpoint of the rarefaction curves, re-sampled to 50,000 reads. Each viral metagenome was categorized according to sample location or depth (epipelagic (epi), <200m; mesopelagic (meso), 200-1000m; bathypelagic (bathy), >1000m; pool, included multiple depths). Error bars indicate standard error of the mean when multiple metagenomes were available for comparison within the specified location and depth category.

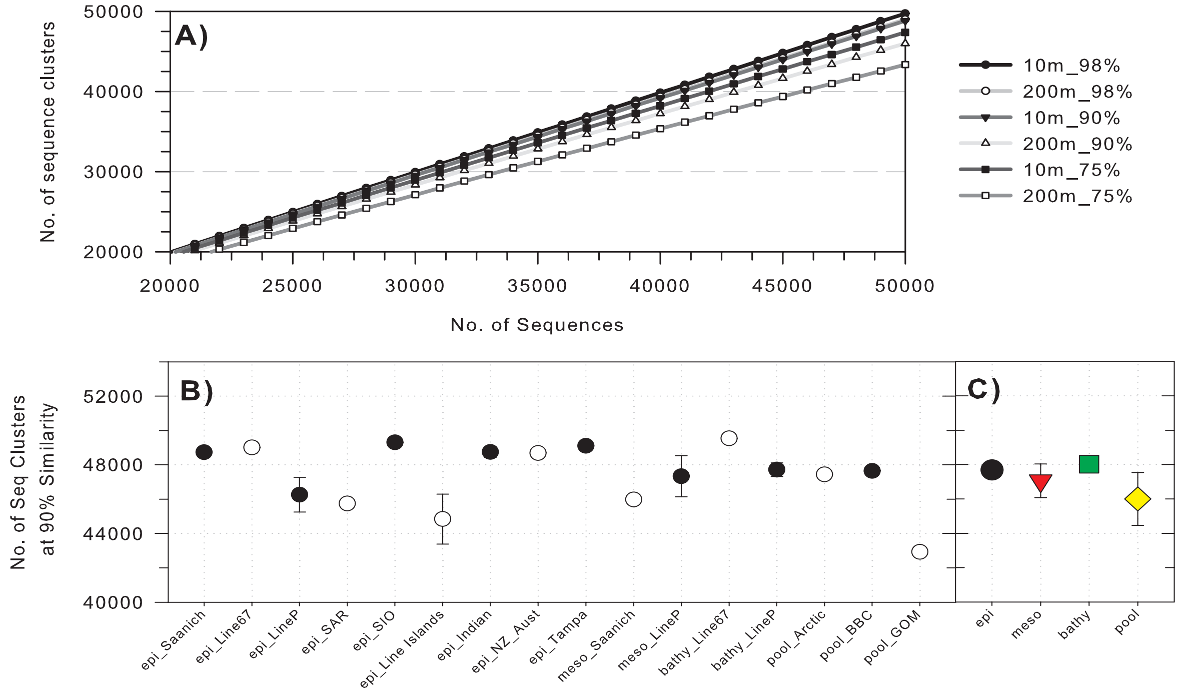

Supplement: Supplementary file 7 [file Image4.PDF]
