# Supplementary material for: Combining genomic sequencing methods to explore viral diversity and reveal potential virus-host interactions
Source: Front Microbiol. 2015 Apr 10;6:265. doi: 10.3389/fmicb.2015.00265 (PMC4392320; doi:10.3389/fmicb.2015.00265)

Figure S5. Cluster analysis of viral diversity for marine viral metagenomes by BLASTn dissimilarity. Saanich Inlet viromes are highlighted in red. Viral metagenomes were abbreviated as found in MetaVir: LXXX = Line P in northeast subarctic Pacific Ocean; MXXX = Line 67 of Monterey, CA; GSXXX = Indian Ocean; SXXX = Scripps Pier in San Diego, CA. For Line P and Scripps Pier viral metagenomes, the final letter in abbreviated name indicates sampling depth: S = 10m, O =100m , A= 500m, D =1300-2000m. For Line 67, depths are as follows: 10m - M1CS, M2MS, M4OS ; 42m – M3MD; 105m – M5OD; 1000m – M6O1K; 4300m – M7O4K.

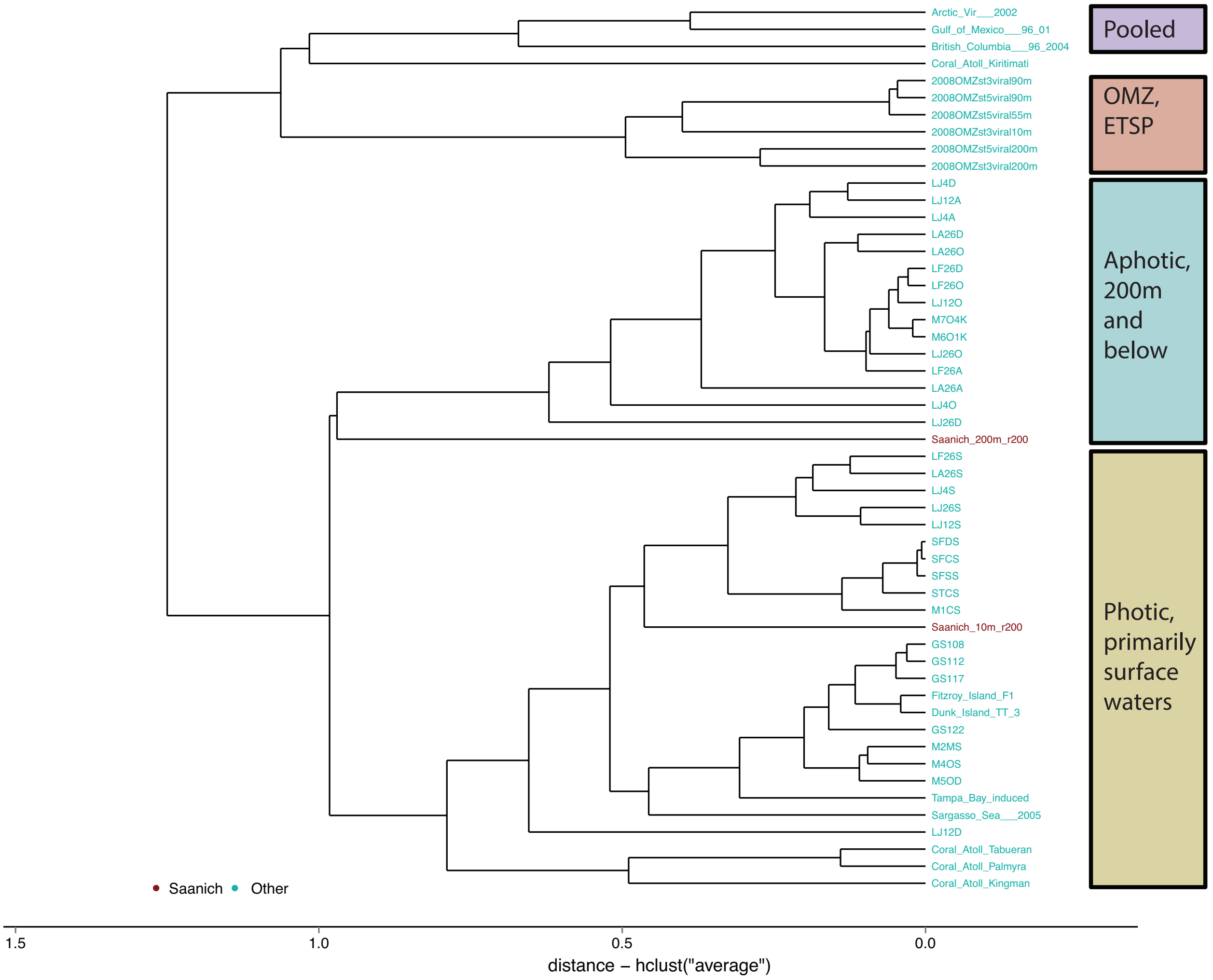

Supplement: Supplementary file 8 [file Image5.PDF]
