# Supplementary material for: Combining genomic sequencing methods to explore viral diversity and reveal potential virus-host interactions
Source: Front Microbiol. 2015 Apr 10;6:265. doi: 10.3389/fmicb.2015.00265 (PMC4392320; doi:10.3389/fmicb.2015.00265)

**Figure S6. Alignment of three fosmids to a pelagiphage genome (HTVC010P) by MAUVE.** The red and green blocks indicate two collinear genomic regions that are internally homologous across all four genome or fosmid sequences. The line inside each colored block indicates the level of similarity within each block for the specified region.

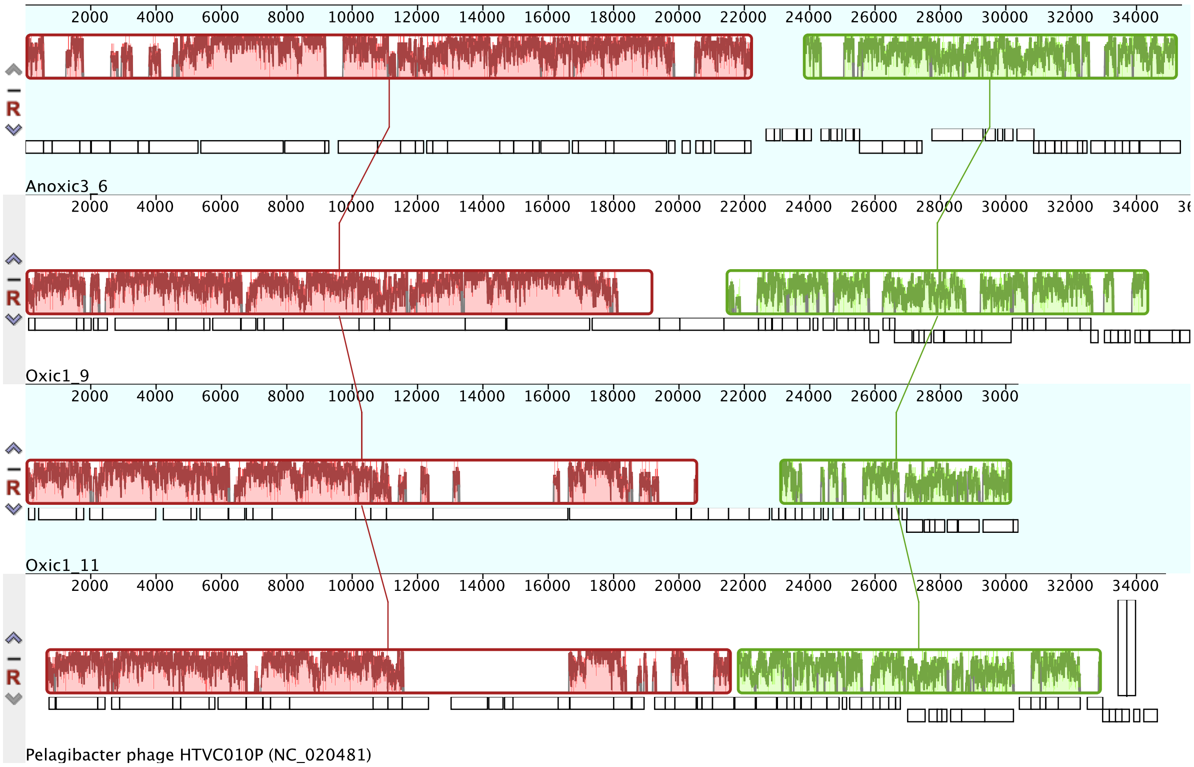

Supplement: Supplementary file 9 [file Image6.PDF]
